# Cardiovascular Events in Patients Received Combined Fibrate/Statin Treatment versus Statin Monotherapy: Acute Coronary Syndrome Israeli Surveys Data

**DOI:** 10.1371/journal.pone.0035298

**Published:** 2012-04-16

**Authors:** Alexander Tenenbaum, Diego Medvedofsky, Enrique Z. Fisman, Liudmila Bubyr, Shlomi Matetzky, David Tanne, Robert Klempfner, Joseph Shemesh, Ilan Goldenberg

**Affiliations:** 1 Cardiac Rehabilitation Institute, The Chaim Sheba Medical Center, Tel-Hashomer, affiliated with the Sackler Faculty of Medicine, Tel-Aviv University, Tel-Aviv, Israel; 2 Leviev Heart Institute, The Chaim Sheba Medical Center, Tel-Hashomer, affiliated with the Sackler Faculty of Medicine, Tel-Aviv University, Tel-Aviv, Israel; 3 The Israeli Society for the Prevention of Heart Attacks, The Chaim Sheba Medical Center, Tel-Hashomer, affiliated with the Sackler Faculty of Medicine, Tel-Aviv University, Tel-Aviv, Israel; 4 Cardiovascular Diabetology Research Foundation, Holon, Israel; 5 Heart Research Follow-up Program, University of Rochester Medical Center, Rochester, New York, United States of America; University of Florida, United States of America

## Abstract

**Background:**

The effect of combination of fibrate with statin on major adverse cardiovascular events (MACE) following acute coronary syndrome (ACS) hospitalization is unclear. The main aim of this study was to investigate the 30-day rate of MACE in patients who participated in the nationwide ACS Israeli Surveys (ACSIS) and were treated on discharge with a fibrate (mainly bezafibrate) and statin combination vs. statin alone.

**Methods:**

The study population comprised 8,982 patients from the ACSIS 2000, 2002, 2004, 2006, 2008 and 2010 enrollment waves who were alive on discharge and received statin. Of these, 8,545 (95%) received statin alone and 437 (5%) received fibrate/statin combination. MACE was defined as a composite measure of death, recurrent MI, recurrent ischemia, stent thrombosis, ischemic stroke and urgent revascularization.

**Results:**

Patients from the combination group were younger (58.1±11.9 vs. 62.9±12.6 years). However, they had significantly more co-morbidities (hypertension, diabetes), current smokers and unfavorable cardio-metabolic profiles (with respect to glucose, total cholesterol, triglyceride and HDL-cholesterol). Development of MACE was recorded in 513 (6.0%) patients from the statin monotherapy group vs. 13 (3.2%) from the combination group, p = 0.01. 30-day re-hospitalization rate was significantly lower in the combination group: 68 (15.6%) vs. 1691 (19.8%) of patients, respectively; p = 0.03. Multivariable analysis identified the fibrate/statin combination as an independent predictor of reduced risk of MACE with odds ratio of 0.54, 95% confidence interval 0.32–0.94.

**Conclusion:**

A significantly lower risk of 30-day MACE rate was observed in patients receiving combined fibrate/statin treatment following ACS compared with statin monotherapy. However, caution should be exercised in interpreting these findings taking into consideration baseline differences between our observational study groups.

## Introduction

Fibrates have been used for the treatment of dyslipidemia (mainly hypertriglyceridemia and low level of HDL cholesterol) for more than 30 years. Their efficacy in reduction of cardiovascular events, particularly in individuals with significant elevations in plasma triglycerides, appears to be well defined [1]–[7]. However, the usefulness of this approach on the background of concomitant statin treatment is unclear. The recently published ACCORD-Lipid trial [8] did not support the addition of one of the fibrates (fenofibrate) to statin therapy in the general population – except for the subgroup of diabetic patients with significant atherogenic dyslipidemia. The effect of the combination of other fibrates with statin on major adverse cardiovascular events (MACE) is unknown. Also, in contrast to statin monotherapy, there are almost no data regarding the effect of fibrates in patients immediately post acute coronary syndrome (ACS).

The main aim of this study was to investigate 30-day rate of MACE in patients participated in the ACS Israeli Surveys (ACSIS) treated on discharge with a fibrate/statin combination (mainly bezafibrate/statin) vs. statin alone. In addition, 30-day re-hospitalization and 1-year mortality rates were also evaluated. In order to assess whether the association between the combined fibrate/statin treatment and clinical outcomes persisted in diverse categories of patients, 30-day MACE rate was determined according to the pre-specified co-morbidities and metabolic status.

## Methods

### Study population

Our patients have been drawn from the ACSIS 2000, 2002, 2004, 2006, 2008 and 2010 enrollment waves. Details of the Acute Coronary Syndrome Israeli Survey (ACSIS) registry have been previously reported [9]. In brief, the ACSIS Registry is a 2-month nationwide survey conducted biennially that prospectively collects data from all ACS admissions in all 25 coronary care units (CCU) in Israel. Patient management was at the discretion of the attending physicians. Eligibility for the study was validated before discharge from the CCU. Discharge diagnoses were recorded as determined by the attending physicians based on clinical, electrocardiographic, and enzymatic criteria. Demographic, historical and clinical data, including medical management, were recorded on pre-specified forms by dedicated study physicians. The Central Data Coordinating Center (based at the Sheba Medical Center) was responsible for the collection of all case report forms and the Israel Heart Society was responsible for keeping the survey database. 30-day outcome rates and 1-year mortality rate were ascertained by hospital chart review, telephone contact and use of the Israeli National Population Registry.

From 2000 to 2010, 11538 consecutive patients with ACS were included in six ACSIS-enrollment waves from the whole country. Our study population comprised 8982 patients who were alive on discharge from the hospital, received statin and for whom 30-day MACE rate was available. Of these, 8545 (95%) patients received statin alone and 437 (5%) received a combined fibrate/statin treatment. In all but 2010 enrollment waves, 1-year follow-up was completed (for 7243 patients).

### Ethics Statement

This register-based analysis of pre-existing data was conducted according to the principles expressed in the Declaration of Helsinki. The ACSIS was approved by the ethical committee of the Sheba Medical Center. All patients provided written informed consent for the collection of data and subsequent analysis.

### Endpoints and definitions

In our study we used pre-specified definitions of the ACSIS. The diagnosis of diabetes was done by the attending physician based on the reported history, medical records and/or for patients with fasting blood glucose of ≥126 mg/dL (7 mmol/L) registered twice or taking any type of pharmacologic antidiabetic treatment prior to enrollment. The diagnosis of hypertension was done based on the reported history, medical records and/or for patients with blood pressure >140/90 mm Hg registered twice, or treatment with antihypertensive drugs prior to enrollment.

Primary endpoint of our study was 30-day MACE rate which was defined as a composite measure of 30-day all-cause mortality, recurrent MI, recurrent ischemia, stent thrombosis, ischemic stroke and urgent revascularization. Secondary endpoints were 30-day re-hospitalization rate and 1-year all-cause mortality.

### Fibrates and statins

In accordance with pre-specified ACSIS forms, data regarding statins and fibrates were collected as the classes of the lipid-lowering medications but not as the specific drugs. We performed specific drugs evaluation among all 437 patients that received fibrate/statin combination. Of them, 405 (92.7%) of patients received bezafibrate, 10 (2.3%) received ciprofibrate and 1 (0.2%) received gemfibrozil (this fibrate is not registered in Israel), whereas in 21 (4.8%) of patients details regarding specific fibrate were unavailable. In regard to statins, 155 (35.5%) of patients received atorvastatin, 257 (58.8%) received simvastatin, 9 (2.1%) received rosuvastatin, 2 (0.5%) received cerivastatin and also 2 (0.5%) received fluvastatin. In 12 (2.7%) of patients details regarding specific statin were unavailable.

### Statistical Analysis

Statistical analysis was performed using SAS statistical software (version 8.2, SAS Institute, Cary, NC). Categorical variables were expressed as percentage, and continuous variables were expressed as mean ± SD. Comparisons of variables were performed by Chi-Square and Fisher's exact test for categorical variables and by unpaired ANOVA test for continuous variables. Survival curves were derived using the Kaplan–Meier approach, and unadjusted comparisons of survival curves were performed using the log-rank test.

In order to determine whether the combination of fibrate & statin vs. statin only treatment on discharge is an independent explanatory variable for 30-day MACE, multivariable logistic regression analysis was applied with adjustment for the following pre-specified variables: age, gender, smoking status, hypertension, diabetes. In addition, the propensity score analysis that includes age, gender, smoking status, hypertension, diabetes, beta-blockers, angiotensin converting enzyme inhibitors and insulin was also calculated.

Interactions between the discharge treatment and potential confounders (categorized by age<65, gender, triglycerides<200, HDL<40, smoking status, presence of diabetes and hypertension) were examined

Results are presented as odds ratio with the appropriate 95% confidence interval. All tests were two-sided and p value<0.05 was considered statistically significant.

## Results

Our population was categorized into 2 groups: 1) patients receiving on discharge statin monotherapy – 8545; and 2) patients receiving on discharge combined fibrate/statin treatment – 437.

### Baseline data

The main clinical and laboratory characteristics of patients are presented in Tables 1 and 2. The majority of patients in both groups were men. Patients from the combination group were younger (58.1±11.9 vs. 62.9±12.6 years). However, they had significantly more co-morbidities (hypertension, diabetes, current smokers) and unfavorable cardio-metabolic profile (with respect to glucose, total cholesterol, triglyceride and HDL-cholesterol levels). Weight, body mass index and blood pressure were also significantly higher in patients of the combination group.

**Table 1:** Baseline characteristics and co-morbidities of the study population.

*Characteristics*	Statin alone (n = 8545)	Fibrate/Statin (n = 437)	p value
Age (years)	62.9±12.6	58.1±11.9	<0.0001
Body mass index (kg/m2)	27.5±4.3	29.5±4.7	<0.0001
Weight (kg)	79.1±14.3	85.4±14.9	<0.0001
Women (%)	1918 (23)	90 (21)	0.365
Hypertension (%)	4829 (57)	296 (68)	<0.0001
Diabetes (%)	2838 (33)	225 (52)	<0.0001
Current smokers (%)	3190 (38)	210 (48)	<0.0001
Past smokers (%)	1654 (20)	85 (20)	0.97
History of stroke	623 (7)	30 (7)	0.73
CRF	796 (9)	48 (11)	0.24
PVD	710 (8.3)	38 (8.7)	0.77

Data are number (%) of patients or mean ± SD.

CRF - chronic renal failure, PVD - peripheral vascular disease.

**Table 2:** In-hospital cardiac interventions and laboratory values.

*Characteristics*	Statin alone	Fibrate/Statin	p value
CABG	396/8526/(5)	17/437/(4)	0.46
PCI	5392/8242/(65.3)	278/419/(75)	0.65
Only angiography	1261/7027/(18)	71/365/(20)	0.47
Systolic blood pressure (mmHg)	142.6±28.3	146.1±25.2	0.01
Diastolic blood pressure (mmHg)	81.8±16.4	84.1±15.4	0.004
Heart rate (beats/min)	80.2±20.0	79.1±16.9	0.27
Glucose (mg/dl)	147.6±73.7	168.2±88.7	<0.0001
Total cholesterol (mg/dl)	189.2±46.2	203.2±48.9	<0.0001
HDL-cholesterol (mg/dl)	41.4±12.7	34.7±8.4	<0.0001
Triglycerides (mg/dl)	157.0±127.2	361.5±262.1	<0.0001
Creatinine (mg/dl)	1.2±0.8	1.1±0.5	0.25

Data are number of patients/number of interventions/(%) for interventions or mean ± SD for laboratory values.

CABG, coronary artery bypass graft; PCI, percutaneous coronary intervantion.

HDL - high density lipoproteins.

Conversion factors for SI units (from mg/dL to mmol/L): for triglyceride multiply by (x) 0.01129, for cholesterol multiply by (x) 0.02586, for glucose multiply by 0.055, for creatinine multiply by (x) 88.4.

No significant differences between the groups were found for history of stroke, chronic renal failure, peripheral vascular disease and in-hospital cardiac interventions.

Data regarding treatment with cardiovascular drugs among the study groups are presented in Table 3. Antiplatelet drugs (aspirin and clopidogrel), beta blockers and angiotensin converting enzyme inhibitors were the most commonly used medications. The use of nitrates, calcium antagonists and diuretics was relatively low. More patients from the combination fibrate/statin group received beta blockers, angiotensin converting enzyme inhibitors and insulin than their counterparts. There were no significant differences in the proportion of patients receiving other cardiovascular drugs.

**Table 3:** Distribution of cardiovascular drugs among the study patients (on discharge).

*Drugs*	Statin alone (n = 8545)	Fibrate/Statin (n = 437)	p value
Aspirin (%)	97	96	0.7
Clopidogrel (%)	72	74	0.2
Beta blockers (%)	82	88	0.004
Nitrates (%)	20	17	0.24
Calcium channel blockers (%)	16	18	0.5
Diuretics (%)	21	22	0.9
Aldosterone antagonist (%)	6	6	0.7
Angiotensin converting enzyme inhibitors (%)	75	80	0.01
Angiotenesin receptor blockers (%)	8	11	0.2
Insulin (%)	7	13	<0.0001

### Outcomes of the study population during follow-up

During the follow-up period of 30 days, development of MACE was recorded in 527 patients: in 513 (6.0%) patients from the statin monotherapy group vs. 14 (3.2%) from the combined fibrate/statin group, p = 0.01 (Table 4).

**Table 4:** Outcomes of the study population during follow-up (crude data).

*Outcomes*	Frequency Missing	Statin alone (n = 8545)	Fibrate/Statin (n = 437)	p value
30-day MACE	-	513 (6.0)	14 (3.2)	0.01
30-day re-hospitalization	-	1691 (19.8)	68 (15.6)	0.03
1-year all-cause mortality	1739	377 (5.5)	11 (3.2)	0.07

Data are number of events/(%).

- The primary endpoint of this study was 30-day Major Adverse Coronary Events (MACE): all-cause mortality, recurrent MI, recurrent ischemia, stent thrombosis, ischemic stroke, urgent revascularization during follow-up.

30-day re-hospitalization rate was also significantly lower in patients from the combination group than in their counterparts from the statin monotherapy group: 68 (15.6%) vs. 1691 (19.8%) respectively, p = 0.03.

Crude 1-year mortality rates in patients of the combination fibrate/statin group tended to be lower than in their counterparts of the statin monotherapy group, but this trend did not reach statistical significance.

**Figure 1:**
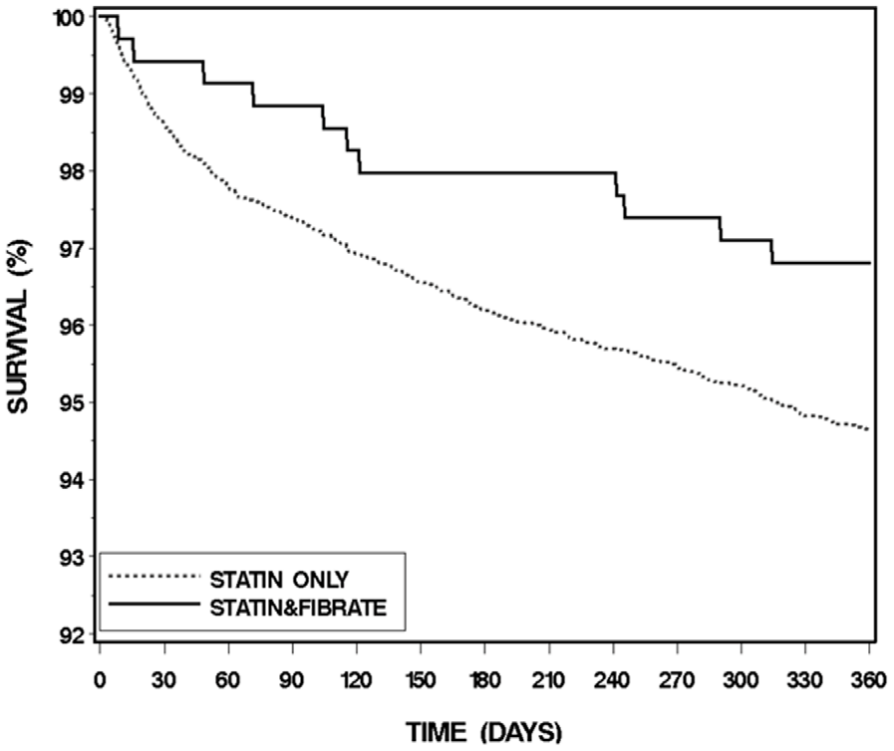
Kaplan-Meier curve of mortality rate during one year follow-up for 7243 patients from years 2000–2008 (combined fibrate/statin therapy vs. statin monotherapy, p log-rank = 0.066).

Kaplan-Meier analysis of 7243 patients from years 2000–2008 (Figure 1) showed that the cumulative probability of survival at 1-year of follow-up (in accordance with the time of occurrence) was numerically higher among patients who received combined fibrate/statin therapy compared with patients who received statin monotherapy, with early separation of event rates between the 2 treatment groups. However, possibly due to sample size limitations related to the relatively low number of mortality events in patients who received combined therapy, these findings did not reach statistical significance (p log-rank = 0.066 for the overall difference during follow-up).

Multivariable analysis (Table 5) identified the combined fibrate/statin treatment as an independent predictor of reduced risk of 30-day MACE following ACS hospitalization with odds ratio (OR) 0.54 [95% confidence interval (CI) 0.32–0.94], corresponding to 46% lower risk. Other significant variables in our model associated with independent risk of MACE during follow-up were female gender and age <65 years. Propensity score analysis has shown results for the effect of the combined fibrate/statin treatment very similar to the multivariable model: OR 0.57, CI 0.33–0.97.

**Table 5:** The applied model of multivariable logistic regression analysis of risk for 30-day MACE in patients immediately post acute coronary syndrome.

*Variables*	Odds Ratio	95% confidence interval	p value
Combined fibrate/statin treatment	0.54	0.32–0.94	0.03
Diabetes Mellitus	1.18	0.98–1.43	0.08
Age <65 years	0.59	0.48–0.73	<0.001
Women	1.45	1.18–1.77	<0.001
Hypertension	1.10	0.90–1.34	0.3
Current smokers	1.04	0.84–1.28	0.7

**Figure 2:**
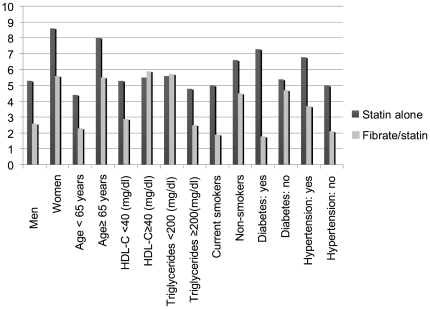
Rate (%) of 30-day Major Adverse Coronary Events (MACE) among the study patients according to the age, gender, level of HDL cholesterol, triglycerides, smoking status, presence of diabetes and hypertension.

In order to assess whether the association between the combined fibrate/statin treatment and the reduction of MACE immediately post ACS hospitalization persisted in diverse categories of co-morbidities and metabolic status, 30-day MACE rate was determined in patients according to gender, age, level of HDL cholesterol, triglycerides, smoking status, presence of diabetes and hypertension (Table 6 and Figure 2). The lower 30-day MACE rate was found in patients receiving combined fibrate/statin treatment, regardless of gender, age, smoking status and hypertension. The beneficial effect of the combined fibrate/statin treatment was augmented in patients with diabetes, low HDL cholesterol and high triglycerides level and was significantly attenuated in patients without diabetes (p-value for combined therapy-by- diabetes interaction = 0.03). In patients with HDL cholesterol ≥40 mg/dl and triglycerides <200 mg/dl the beneficial effect of the combination fibrate and statin was absent. However, no statistically significant interactions were identified in these subsets.

**Table 6:** Effect of combined fibrate/statin treatment vs. statin monoterapy on 30-day Major Adverse Coronary Events (MACE) in risk subgroups: odds ratio and 95% confidence interval (CI).

*Risk Subgroup*	Odds Ratio (95% CI)	P-value for interaction
**Gender**		
Men	0.48 (0.25–0.94)	0.7
Women	0.62 (0.25–1.57)	
**Age**		
Age <65 years	0.51 (0.24–1.09)	0.6
Age ≥65 years	0.66 (0.31–1.43)	
**HDL-C**		
HDL-C <40 (mg/dl)	0.53 (0.26–1.09)	0.2
HDL-C ≥40 (mg/dl)	1.08 (0.43–2.7)	
**Triglycerides**		
Triglycerides <200 (mg/dl)	1.03 (0.45–2.36)	0.2
Triglycerides ≥200(mg/dl)	0.52 (0.24–1.14)	
**Atherogenic dyslipidemia** (Triglycerides >200 & HDL-C<40)		0.18
Atherogenic dyslipidemia: yes	0.35 (0.16–0.75)	
Atherogenic dyslipidemia: no	0.89 (0.41–1.93)	
**Smoking status**		
Current smokers	0.37 (0.14–1.0)	0.3
Non-smokers	0.66 (0.35–1.26)	
**Diabetes Mellitus**		
Diabetes: yes	0.23 (0.08–0.62)	0.03
Diabetes: no	0.87 (0.46–1.67)	
**Hypertension**		
Hypertension: yes	0.53 (0.29–0.98)	0.7
Hypertension: no	0.42 (0.13–1.32)	

HDL-C - high density lipoproteins cholesterol.

## Discussion

The main finding of our study is lower risk of 30-day MACE rate in the patients that received combined fibrate/statin treatment following ACS compared with the statin monotherapy. During this period re-hospitalization rate was also significantly reduced in patients receiving both drugs. One-year mortality rate in patients from the combined fibrate/statin group tended to be lower than in their counterparts from the statin monotherapy group; however this trend did not reach statistical significance.

Statins use is clearly efficacious in the treatment and prevention of coronary artery disease, particularly in ACS [10]–[15]. As a result, currently nearly all patients with ACS receive prescriptions for statin in developed countries, unless contraindicated. However, despite of this almost ubiquitous use of statins, a significant number of recurrent MACE still occur and many patients remain at high residual cardiovascular risk. Due to their beneficial effects on lipid metabolism (mainly a decrease in triglyceride and an increase in HDL-cholesterol), fibrates are good potential candidates for reducing this residual cardiovascular risk in patients with atherogenic dyslipidemia. Currently only two fibrates (bezafibrate and ciprofibrate) are registered in Israel; of them, bezafibrate is the most widely prescribed fibrate which is used in the vast majority of the cases.

Although less clinical interventional studies have been performed with fibrates than with statins, the therapeutic benefit using one of the three “major" fibrates (fenofibrate, bezafibrate and gemfibrozil) was demonstrated among patients with high triglycerides and low HDL-cholesterol. In contrast, in patients without dyslipidemia the favorable effects of fibrates on the “hard" cardiovascular end points were absent and usually there were no significant difference between fibrate and placebo groups [16]. For example, in a meta-analysis of dyslipidemic subgroups from the five main fibrates trials, a 35% RR reduction in cardiovascular events was observed compared with a 6% RR reduction in those not meeting dyslipidemic criteria [17]. As expected, in a so called “general population" – reflecting a blend of effects in patients with and without atherogenic dyslipidemia [18] – the beneficial effect of fibrate therapy was diluted, producing only a modest 10% RR decrease in major cardiovascular events and a 13% RR reduction in coronary events in the other meta-analysis [19].

Nevertheless, in a time of a near ubiquitous presence of statin treatment in the patients with cardio-vascular atherosclerotic diseases or high risk for them, an appropriate question is: “What is the role of fibrates as an addition to statin-based therapy?" The ACCORD Lipid study had shown in a prespecified subgroup analysis (941 out of 5518 patients) that patients with atherogenic dyslipidemia benefited from the addition of fenofibrate to simvastatin. Among all other 4548 patients included in this analysis (without atherogenic dyslipidemia) such rates were 10.1% in both fenofibrate and placebo study groups [8].

In line with previous studies, our data suggest that the favorable effect of fibrates added to statin is particularly noticeable in patients with diabetes, low HDL cholesterol and high triglycerides level. Therefore, in appropriate patients fibrates probably may lead to cardiovascular risk reduction not only as monotherapy but in combination with statins as well. On the other hand, in patients without atherogenic dyslipidemia a beneficial effect of the combined fibrate/statin treatment on 30-day MACE rate was absent.

A meta-analysis by Briel et al. [20] suggested that simply increasing the amount of circulating high density lipoprotein cholesterol does not reduce the risk of coronary heart disease events. This meta-analysis did not take into consideration the specific sort of intervention that alters levels of high density lipoprotein cholesterol. Obviously, different medications or life style changes may have different impacts on cardiovascular risk. Particularly, our group has shown previously using the Bezafibrate Infarction Prevention (BIP) trial data that that HDL-C level-raising therapy with bezafibrate is associated with long-term mortality reduction that may be related to the degree of HDL-C response to treatment [21]. These results are consistent with the similar analyses from the other fibrates studies [22], [23]. Circulating HDL particles are greatly heterogeneous with a very complex metabolic profile. HDL-C measures the cholesterol content of nascent HDL, HDL2, and HDL3 particles and is, therefore, a crude marker of reverse cholesterol transport. There is a broad agreement that reverse cholesterol transport, the process of transporting excess cholesterol from the arterial wall's foam macrophages to the liver, bile, and feces is one of HDL's important antiatherogenic properties [24].

Also the triglyceride -rich environment has been shown to be strongly associated with an atherogenic lipoprotein phenotype or atherogenic dyslipidemia. The atherogenic, triglyceride -rich lipoprotein environment is common to insulin resistance, obesity, metabolic syndrome, prediabetes, diabetes and usually is associated with low high-density lipoprotein cholesterol (HDL-C) and higher CVD risk [25]. Fibrates, in brief, via peroxisome proliferator-activated receptors (PPAR)-α, mediate the transcriptional regulation and expression of genes involved in lipid metabolism reducing the generation of atherogenic remnants, promotes β-oxidation of fatty acids and decrease fatty acid synthase activities; increase lipolysis and plasma clearance of atherogenic lipoproteins [7]. In patients with atherogenic dyslipidemia (high triglycerides and low HDL-cholesterol) fibrates were consistently associated with reduced risk of cardiovascular events [16].

In contrast to statins, the impact of fibrates on cardiovascular events in the setting of ACS is unclear. Only one small study suggested that bezafibrate was associated with a lower incidence of major cardiovascular events during hospitalization [26]. The role of fibrates in the possible MACE reduction following ACS hospitalization is unknown. Therefore, our data offer essential insight on this gape of knowledge.

### Study Limitations

Our study has several important limitations. Data regarding alcohol consumption and medications dose and frequency were not available.

Although the present study assessed the impact from addition of fibrate to statin on clinical outcomes within a pre-specified setting of prospective nationwide surveys, it was not a randomized controlled trial and we cannot rule out other factors that could have influenced the observed improvements in clinical outcomes. It should be mentioned that the baseline characteristics of the two study groups were different. Particularly, larger proportion of patients in the combined therapy group had poorer lipid profile. These systematic differences could lead to the observed differential treatment effect.

Multivariable and interaction analyses were utilized for MACE rate evaluation in an aim to address the lack of randomization and baseline differences between groups that may confound the results. However, caution should be used in interpreting our findings which require confirmation in prospectively planned controlled clinical trials. Furthermore, the lack of interaction in some subsets that were assessed could be due to a relatively low number of observed MACE cases.

In conclusion, a significantly lower risk of 30-day MACE rate was observed in patients receiving combined fibrate/statin treatment (mainly bezafibrate/statin combination) following ACS compared with statin monotherapy, with a more pronounced effect among those with diabetes.
